# *Clostridium baratii*: a rare case of pneumonia associated with an Alzheimer patient in Rio de Janeiro, Brazil

**DOI:** 10.1099/jmmcr.0.005041

**Published:** 2016-08-30

**Authors:** Carla Ormundo Gonçalves Ximenes Lima, Vinicius Magno da Rocha, Eliane de Oliveira Ferreira, Joaquim Santos Filho, Lucia Rodrigues Serradas, Rodrigo Otávio Silveira Silva, Francisco Carlos Faria Lobato, Regina Maria Cavalcanti Pilotto Domingues

**Affiliations:** ^1^​ Hospital de Clínicas Rio Mar Barra LTDA, Avenida Candido Portinari, 555. Rio de Janeiro, RJ, Brazil; ^2^​ Hospital Universitário Gaffrée e Guinle, Rua Mariz e Barros, 755. Rio de Janeiro, RJ, Brazil; ^3^​ Universidade Federal do Rio de Janeiro, Laboratory of Anaerobes Biology, Avenida Carlos Chagas Filho, 373, Bloco I-2. Rio de Janeiro, RJ, Brazil; ^4^​ Universidade Federal do Rio de Janeiro, Polo Xerém, Estrada de Xerém, 27. Duque de Caxias, RJ, Brazil; ^5^​ Universidade Federal de Minas Gerais, Veterinary School, Avenida Antônio Carlos, 6627. Belo Horizonte, MG, Brazil

**Keywords:** *Clostridium baratii*, pneumonia, Alzheimer, Brazil

## Abstract

**Introduction:**

*Clostridium baratii* is rarely associated with human diseases. Infection is usuallcaused by ingestion of contaminated food, and infant botulism is the most common clinical presentation.

**Case Report:**

Here we report a case of pneumonia by a non-toxigenic strain of *C. baratii* in an Alzheimer 70-year-old male with sepsis in Rio de Janeiro, Brazil. The micro-organism was identified by phenotypical tests, mass spectrometry (MALDI-TOF), DNA amplification (PCR) and sequencing of the 16S rRNA gene. Testing for the presence of botulinum F toxin was made using multiplex PCR. Bioassay for a large number of colonies was performed in mice to evaluate the production of any lethal toxin, but the results were negative.

**Conclusion:**

To our knowledge, there are no cases of *C. baratii* infection reported in Brazil and we highlight the importance of anaerobic lab tests in the standard routine of diagnosis.

## Introduction

The genus *Clostridium *gathers anaerobic Gram-positive rods that are capable of forming endospores. The majority of members of this genus are strict anaerobes, but there are considerable species variations with respect to oxygen toxicity (Krieg *et al.*, 2010). There are at least 150 species described, most of them are saprophytic and less than 20 species are related to human diseases (Shu *et al.*, 2008). Although members of the genus are usually found as microbiota of the human intestinal tract, their disease spectra in humans are considered to be broad (Hannett *et al., *2014). *Clostridium baratii* is an obligately anaerobic bacterium that is rarely encountered as causing human infections (Hannett *et al., *2014). In 1985 the first report of botulism neurotoxin production by a strain of this organism was made (Hall *et al.*, 1985). Hannett *et al.* (2014) have described two cases of F botulism infection in elderly patients, 79- and 68-years-old, both with history of diplopia and weakness. Since both cases were clustered together in time and geography, molecular typing was performed and proved that they were unrelated genetically. In 2015, three cases of food-borne botulism by the consumption of Bolognese sauce were reported in France (Tréhard *et al.*, 2016). In this case, strains were isolated from stools and the authors proved the meat used to make the sauce to be the contaminant.

*C. baratii* producing type F neurotoxin is a potential human pathogen and some severe cases, such as a 3-year-old boy with Kawasaky syndrome (Iaria *et al.*, 2007), a lung abscess in a patient with an invasive pulmonary aspergillosis (Shu *et al.*, 2008), and a liver abscess in a healthy adult (Huang *et al.*, 2012) have been reported over the years. Here we report a case of pneumonia by a non-toxigenic strain of *C. baratii* in an Alzheimer 70-year-old male with sepsis in Rio de Janeiro, Brazil.

## Case report

In 2014, a 70-year-old man was admitted to a private hospital with complaints of asthenia and night fever. Empirical treatment of urinary tract infection was in progress with ciprofloxacin 500 mg twice a day outpatient, with no clinical answer. The patient also had previous diagnoses of chronic kidney disease, benign prostatic hyperplasia, chronic ischemic stroke and Alzheimer’s disease.

**Fig. 1.**
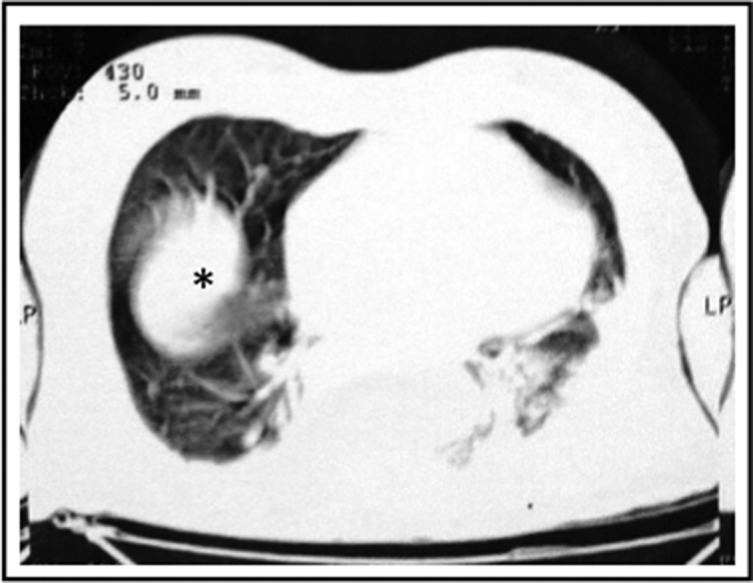
Computed tomography of the abdomen showing a mass with fluid-retaining in the central and left cavity of the lung, and thick irregular wall over the right and left lung (LP). A dense mass is shown in the center of right lung (∗).

On admission, pulmonary and abdominal computed tomography (CT) scans were performed. Left inferior lobe consolidation and a small pleural bilateral effusion (Fig. 1) were identified. There was also a fecaloma, promoting abdominal distention.

The primary diagnosis was lung sepsis, and the patient was admitted to an intensive care unit (ICU). Urinalysis, urine culture, haemogram, haemoculture and C-reactive protein (CRP) dosage were made. A few hours after ICU admission, empiric antibiotic therapy was started. The initial regimen of antibiotic was piperacillin-tazobactam 3.375 g intravenously three times a day. After seven days on this regimen, the patient still presented a high leucocytes count and increasing levels of CRP. Unfortunately, both blood and urine cultures were negative. Facing this clinical scenario, the medical team chose to change antibiotic and perform another pulmonary CT scan. Empirically, meropenem 1 g twice a day was started to replace piperacillin-tazobactam. The latest pulmonary CT scan showed a worsening of pleural effusion and intense pleural bleeding in the left lung. Despite the change of the antibiotic regimen, and its use for a week, clinical improvement was not observed. The patient still had a fever, respiratory distress and haemodynamic instability. At this point, another diagnostic measure was attempted: a surgical pleuroscopy. During this procedure, both pleural effusion and its fragments were obtained for microbiology screening.

Materials obtained by pleuroscopy were inoculated on blood agar plates (5 % desfibrinated sheep blood), chocolate agar with Vitox (PlastLabor) and in a BD BACTEC anaerobic test bottle (Becton Dickinson). All plates were incubated for 48 h at 37 °C, under aerobic and capnofilic atmosphere. The blood culture was positive seven days after inoculation. Gram stain of this culture revealed the presence of Gram-positive spore-forming rods. The flask was sent to the Laboratory of Anaerobes Biology at Universidade Federal do Rio de Janeiro (UFRJ), where it was inoculated on 5 % sheep blood agar plates supplemented with 5 mg l^−1^ haemin and 1 mg ml^−1^ menadione. The plates were incubated in the COY glove box (Coy Laboratory) and revealed translucid, β-haemolytic, smooth, circular, yellow-pigmented colonies after incubation for 48 h. Two analyses were conducted for the identification of the sample: (i) phenotypical tests (Table 1) (Jousiemies-Somier *et al.*, 2002) and (ii) mass spectrometry (MS) by MALDI-TOF BioTyper System (Bruker Daltonics). Tests indicated a strain of *C. baratii. *Additionally antimicrobial susceptibility patterns and MIC values were determined by the ETESTstrips (bioMérieux) and were used to breakpoints standardized by the Clinical and Laboratory Standard Institute (CLSI, 2014). For quality control, clinical isolates, namely *Bacteroides fragilis* ATCC 25285 and *Staphylococcus aureus* ATCC 29213, were used in parallel for each incubation. The following antibiotics were tested: vancomycin (MIC 0.38 µg ml^−1^ – sensitive), metronidazole (MIC 0.30 µg ml^−1^ – sensitive), clindamycin (MIC 0.38 µg ml^−1^ – sensitive), ertapenem (MIC 0.006 µg ml^−1^ – sensitive) and imipenem (MIC 0.094 µg ml^−1^ – sensitive).

**Table 1.** Characteristics of Nagler-positive species of the genus *Clostridium*

	Species			
	*C. perfringens*†	C. baratii*†	*C. bifermentans*†	*C. sordellii*†
Indole	−	−	+	+
Reverse-CAMP test	+	−	−	−
Fermentation of:				
Mannose	+	+	−	−
Xylose	−	+	−	−
Arabinose	−	+	−	−
Glucose	+	+	+	+

*C. baratii phenotype tests performed in this study.

†Characteristics of Gram-positive spore-forming bacilli according to the Wadsworth Anaerobic Bacteriology Manual, 5th edn.

Genotypic characterization was done in the Veterinary School of the Universidade Federal de Minas Gerais. Total bacterial DNA was extracted using the commercial kit QIAamp DNA mini kit (QIAGEN) according to the manufacturer’s instructions. Amplification and sequencing of the 16S rRNA gene were performed according to a previously described method (Fox *et al.*, 2011). PCR products were purified using a Wizard PCR Preps DNA purification system (Promega) and sequenced using forward and reverse primers. Sequencing reactions were performed using a BigDyeTerminator v3.1 cycle sequencing kit (ThermoFisher) and run on an ABI 3730XL genetic analyzer (ThermoFisher). The 16S rRNA gene sequences were compared with those of reference strains in the GenBank database of the National Center for Biotechnology Information (http://ncbi.nlm.nih.gov) using the BLASTN computational tool and nucleotide sequence identity ≥98 % was used as the criterion for species identification. Together, all tests showed that the organism was *C. baratii*. The detection of botulinum toxin using PCR (De Medici *et al.*, 2009) and bioassay in mice (Sebald & Petit, 1997) was negative.

After diagnosis, metronidazole 500 mg three times a day intravenously was started and within 24 h the patient had no more fever. One week later, chest radiographs and clinical symptoms had improved.

## Discussion

Few cases of infection due to *C. baratii* have been previously described. The first case reported was a bacteraemia by *C. baratii *associated with Kawasaki syndrome in a 3-year-old child (Iaria *et al.*, 2007). Shu *et al*. (2008) described a lung abscess superimposed on invasive pulmonary aspergillosis in a 47-year-old man previously diagnosed with myelodysplastic syndrome. Huang *et al*. (2012) isolated *C. baratii* in a liver abscess caused by a cholecystitis emphysematous in a healthy adult. Recently, two cases of adult botulism caused by botulinum neurotoxin-producing *C. baratii* were reported in patients from the same geographical region. The pathophysiology hypothesis accepted in both cases was intestinal toxemia-related disease (Hannett *et al.*, 2014).

Severe pneumonia described here is a clinical manifestation never before related to *C. baratii*. Moreover, diseases caused by *Clostridium *are usually life-threatening conditions, making imperative the prompt diagnosis for successful treatment (Bischoff, 2012). However, the low suspicion of both clinicians and bacteriologists in Brazil concerning commensal micro-organisms as responsible for infection hampers diagnosis. Furthermore, specifically in the case of uncommon micro-organisms, besides the sampling and proper preparation of the patient’s materials, the diagnostic strategy should apply specific and effective methods (Ngo *et al.*, 2013; Park *et al.*, 2009; Tsybuliak & Epifanov, 2009). In our report, at the first medical approach, although samples had been collected, the identification attempt failed. Probably the sampling and/or material preparation were inappropriate. Only after site-specific sampling (pleural effusion and tissue fragments collected by pleuroscopy) and proper preparation of the materials, was isolation of rod-shaped anaerobic Gram-positive cultures possible.

In this current reported case, we isolated a *C. baratii* non-toxigenic strain but it still caused a severe pulmonary infection. The patient was an Alzheimer 70-year-old male with several complications and probably underwent therapies or procedures that could have perturbed the normal flora andled to colonization of *C. baratii. *Even though this micro-organism is usually related to intestinal toxemia and infant botulism, in this patient it could have led to a pulmonary infection. This scenario highlights the importance and possible role of commensal anaerobes in human diseases, reinforcing the need for appropriate anaerobic research on biological samples.
